# Mediterranean Diet: Lipids, Inflammation, and Malaria Infection

**DOI:** 10.3390/ijms21124489

**Published:** 2020-06-24

**Authors:** Adriana R. Silva, Bianca P. T. Moraes, Cassiano F. Gonçalves-de-Albuquerque

**Affiliations:** 1 Laboratório de Imunofarmacologia, Instituto Oswaldo Cruz, Fundação Oswaldo Cruz (FIOCRUZ), Rio de Janeiro 21040-900, Brazil; biancapt@gmail.com; 2 Programa de Neurociências da Universidade Federal Fluminense (UFF), Niterói 24020-141, Brazil; 3 Programa de Pós-Graduação em Biologia Celular e Molecular, Instituto Oswaldo Cruz, FIOCRUZ, Rio de Janeiro 21040-900, Brazil; 4 Laboratório de Imunofarmacologia, Universidade Federal do Estado do Rio de Janeiro (UNIRIO), Rio de Janeiro 20210-010, Brazil; 5 Programa de Pós-Graduação em Biologia Molecular e Celular, UNIRIO, Rio de Janeiro 20210-010, Brazil

**Keywords:** mediterranean diet, fatty acid, omega-3, omega-6, omega-9, inflammation, malaria

## Abstract

The Mediterranean diet (MedDiet) consists of consumption of vegetables and healthy oils and have beneficial effects on metabolic and inflammatory diseases. Our goal here is to discuss the role of fatty acid content in MedDiet, mostly omega-3, omega-6, and omega-9 on malaria. Malaria affects millions of people around the globe. The parasite Plasmodium causes the disease. The metabolic and inflammatory alterations in the severe forms have damaging consequences to the host. The lipid content in the MedDiet holds anti-inflammatory and pro-resolutive features in the host and have detrimental effects on the Plasmodium. The lipids from the diet impact the balance of pro- and anti-inflammation, thus, lipids intake from the diet is critical to parasite elimination and host tissue damage caused by an immune response. Herein, we go into the cellular and molecular mechanisms and targets of the MedDiet fatty acids in the host and the parasite, reviewing potential benefits of the MedDiet, on inflammation, malaria infection progression, and clinical outcome.

## 1. Introduction

Over the years, many scientific articles, newspapers, and magazines have promoted the beneficial role of the Mediterranean diet (MedDiet), including association with a decreased incidence of cardiovascular events, diabetes, metabolic syndrome, and breast cancer [1,2,3,4,5].

The MedDiet is a food pattern of countries bordering the Mediterranean Sea, a “Mediterranean lifestyle” with three primary components—olive oil, wine, and bread. The MedDiet dietary pattern includes high consumption of fruits, vegetables, legumes, olive oil, and whole grains, moderate-to-high intake of fish (and seafood), moderate alcohol intake, and low consumption of meat products, dairy and sweets [6,7,8,9] (Table 1). MedDiet has low total fat (<30%), low saturated fat (<10%), and high monounsaturated fatty acid:saturated fatty acid (MUFA:SFA) ratio [8]. Polyunsaturated fatty acid (PUFA) composes the MedDiet, and includes the omega-6 linolenic acid (LA) and marine-derived omega-3 fatty acids, such as α-linolenic acid (ALA), eicosapentaenoic acid (EPA), docosapentaenoic acid (DPA), and docosahexaenoic acid (DHA). Mediterranean seafood species are a dietary source of omega-3 fatty acids, particularly the pelagic fish species [10], but plants and dry fruit might provide a reasonable amount of omega-3 fatty acids. The main source of MUFA is the olive oil [11]. Lipofilic vitamins sources include fish (vitamin D), vegetable and fruits, and minor grade dairy products (vitamins E and A) [12].

Olive oil and fish stand out as health food and the lipid composition of omega-9 and omega-3 fatty acids can account for the wide variety of beneficial effects [1,5,17]. The amount of lipids in the MedDiet is the same as the occidental diet, and the key difference is the type of lipid with a higher amount of unsaturated fatty acid in the MedDiet. Many reports describe the effects of unsaturated fatty acids modulating both inflammation and metabolism, denoting the benefits of the MedDiet [18,19].

**Figure 1:**
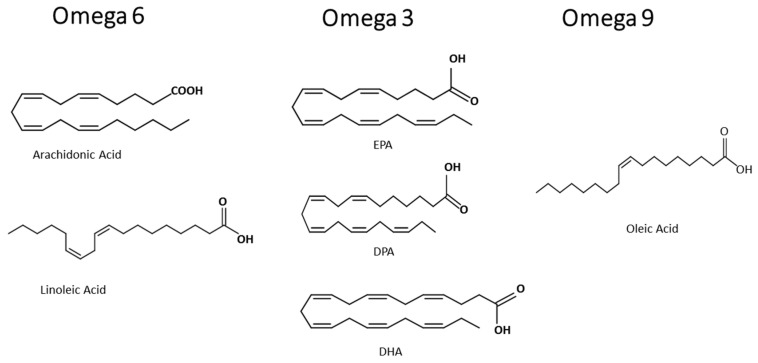
Structures of typical omega-6, omega-3, and omega-9 fatty acids.

Besides the anti-inflammatory effects of fatty acids, the pro-resolutive role of fatty acids are related to the control of the development of inflammatory, infectious, and parasitic diseases. The aim of this review is to summarize the effects of omega-3 eicosapentaenoic acid, docosahexaenoic acid and docosapentaenoic acid, the omega-6 arachidonic acid, and the omega-9 oleic acid, on immune cells (structures in Figure 1), preventing exacerbated inflammation and its deleterious effect, and point out that the control of activation of immune cells and some intracellular signaling proteins and transcription factors are the molecular mechanisms of the beneficial role of these fatty acids. As fatty acid participation in malaria is not broadly reviewed and understood, we both discuss the modulation of parasite fatty acid metabolism; and host diet supplementation with the fatty acid components of MedDiet, relating that to the control of the host response leading to a better outcome. There is a high demand for the discovery of adjuvant therapies or approaches as diet supplementation for parasitic disease management, prevention of severe malaria symptoms, and the improvement of clinical handling.

## 2. Malaria and Inflammation

### 2.1. Malaria Cycle, Vaccine, and Treatment

Malaria has gone through tireless efforts to eradicate [20], and besides the advances in diagnosis and treatment, it remains one of the most severe and life-threatening infectious diseases [21]. The parasite plasmodium causes malaria. Both *Plasmodium falciparum* and *vivax* are the most prevalent in Africa (99.7%) and in America (74.1%), affecting 219 million people in 2017, where young children in sub-Saharan Africa are the most affected [22].

Approximately 40 species of the mosquito genus Anopheles are considered to be 41 dominant vector species (DVS) of the human malaria parasite [23]. Research mainly focuses on preventing mosquito infection with plasmodium, and preventing transmission of the disease [24]. The long-lasting insecticide-treated bed nets and indoor residual spraying is responsible for decreasing malaria infection in Africa between 2000 and 2015 [25].

During the mosquito–human life cycle, the Plasmodium undergoes many morphological states [26], and during the symptomatic phase, the parasites might replicate exponentially in patients. Female Anopheles mosquito transmits sporozoites, that reach the bloodstream and migrate to hepatocytes. On hepatic cells, the parasite multiplies generating merozoites. Merozoites enter into the bloodstream infecting red blood cells (RBC), starting the intra-erythrocytic cycle. A small part of the schizogony generates the gametocytes required for transmission to the mosquito [21,26,27].

There is no effective malaria vaccine [24]. The only registered malaria vaccine confers only short-term and modest protection [28]. BCG-vaccinated volunteers presented a higher frequency of moderate or severe clinical symptoms than control. It also generates an earlier natural killer cell and monocyte activation, which might have activity against heterologous pathogens [29]. Malaria treatment also remains a challenge. Artemisinin-based combination therapies are the gold standard treatment, when correctly employed [24]. Considerable effort has been made for the development of new malaria treatment as the potential use of “metacaspases”, the unique proteases absent in humans [30]. Tafenoquine was recently registered to treat *Plasmodium vivax*. In a controlled trial in Ethiopia, Peru, Brazil, Cambodia, Thailand, and the Philippines, a single-dose of tafenoquine reduced the risk of *Plasmodium vivax* recurrence in patients with regular glucose-6-phosphate dehydrogenase activity [31].

### 2.2. Malaria Physiopathology

Malaria is classified into uncomplicated and complicated malaria. The first one can be asymptomatic or have mild signs, like fever, with no organ dysfunction [32]. The last one is caused by an inadequate immune response, presenting fever, organ dysfunction, respiratory distress, hepatorenal failure, cerebral malaria, shock, and death [32,33]. In this regard, an immune response is linked to the severity of malaria as well as sepsis. The immune checkpoint blockade is a striking success in specific diseases, suggesting that it would be useful for preventing and treating a range of infectious diseases [34].

A balance between host pro-inflammatory and anti-inflammatory immune responses is a crucial determinant for the pathogenesis of severe malaria. Weaker pro-inflammatory responses would confer protection against inflammation-driven organ damage, but could allow parasite persistence and proliferation, while exuberant pro-inflammatory responses are predicted to control the infection but due to maladapted inflammatory responses could trigger lethal immunopathology, including cerebral malaria [35,36,37]. Controlling deleterious excessive immune response is a strategy to obtain desired therapeutic or preventive strategies in cerebral malaria [38].

The host immune system recognized parasite molecules during infected erythrocyte rupture by the receptor, such as the toll-like receptors (TLRs), triggering the production of pro-inflammatory mediators [35]. Parasite factors stimulate the synthesis and release of cytokines, such as tumor necrosis factor-alpha (TNF-α), interleukin (IL)-6, and IL-1 by macrophages and other cells, resulting in fever, chills, and hyperkinetic hemodynamic changes [39].

The glycosylphosphatidylinositol (GPI) of *Plasmodium falciparum* is a malaria pathogen-associated molecular pattern (PAMP) and a toxin. Purified GPI induces the expression of pro-inflammatory cytokines TNF-α, IL-1, and IL-12 [40], the inducible nitric oxide synthase (iNOS or NOS2) [41], and adhesion molecules on the endothelium, increasing endothelial-cell binding by parasitized red blood cells (pRBC) [42]. GPI alone is sufficient to induce malarial shock-like syndrome [40]. The proinflammatory cytokine IL-1α has a role in the liver pathology of the experimental malaria model. *Plasmodium chabaudi* recruits IL-1α-producing neutrophils to the liver. The IL-1α production, independent of the nucleotide-binding oligomerization domain, Leucine-rich Repeat, and Pyrin domain containing 3 (NLRP3) inflammasome activation, increases the production of TNF-α, causing inflammation and hepatocytes apoptosis [43]. A recent report showed the dependence of reactive oxygen species (ROS) produced by xanthine oxidase, to induce the production of inflammatory cytokine in primary human monocyte-derived macrophages in response to plasma patients with cerebral malaria [44]. Infections with intestinal parasites did not alter the patterns of cytokine production in individuals from populations living with endemic malaria, when infected with *Plasmodium falciparum* and *Plasmodium vivax* [45]. This finding differed from other studies where children with malaria coinfected with Gram negative bacteria, presented a lower parasite burden, with production of IL-4, IL-10, IL-12, and interferon-gamma (IFN-γ) lowering parasitemia, without worsening anemia [46].

### 2.3. The Role of Monocytes in Malaria

The hallmark of *Plasmodium falciparum* infection is exponential parasite growth, with a strong host immune response and microvascular obstruction, due to the adherence of mature parasites to the endothelium, leading to a life-threatening condition [47]. During the blood-stage of malaria infection, the inflammatory response is amplified, with cell activation and pro-inflammatory mediator production, by reciprocal activation loops of white blood cells and endothelial cells [48]. During severe malaria, besides cytokine production, there is an increase in adhesion molecules expression, release of angiopoietin 2 (Ang-2), decrease of nitric oxide (NO), and adhesion of parasitized RBC (pRBC) and leukocytes to brain vasculature, causing microvascular dysfunction that ends up in an activation of microglial cells and astrocytes [49]. Monocytes are vital to control plasmodium burden and to protect the host from the infection because they are associated with pathogenesis and drive the inflammation and sequestration of pRBC in the brain [27]. Monocytes do not phagocytose erythrocytes infected with mature gametocytes [50], but they phagocytize merozoites and asexual pRBC [27]. Opsonized Plasmodium with specific IgG enhances the phagocytic activity and offers protection against malaria infection [51,52,53,54].

Plasmodium-specific IgE and activated monocytes have a role in disease severity [55]. Ortega-Pajares and Rogerson, 2018 reviewed the role of “primed” monocytes at the molecular level in malaria infection in a mechanism called trained immunity in which monocytes have “memory” [27]. In the experimental malaria model in mice, monocytes are the main sequestered leukocyte, inducing and aggravating brain inflammation by recruiting CD4^+^ and CD8^+^ T cells [56,57] and also by the chemokine (C-X-C motif) ligand 10 (CXCL10) production [57].

Recent work used a model to predict parasite multiplication rate, and host response in subjects infected with *P. falciparum*. The authors predicted that parasite-growth inhibition was associated with a lower expression of CXCL10 and IFN-α-associated genes [58]. These results were consistent with findings that CXCL10 deletion and neutralization decreases the parasite load in mice [57]. Hence this model could be a useful tool to study malaria infection course.

In an experimental cerebral malaria study, the induction of IL-33 has led to the polarization to the pro-resolutive macrophages of M2 phenotype and forkhead box P3 (Foxp3) Treg cells [59]. Additionally, IL-33-treated mice presented a reduction in parasitemia at the initial step of infection, but they presented hyperparasitemia and died at the late stages of infection. IL-33 is a new player in experimental cerebral malaria development because the microvascular pathology was dependent on IL-33/ST2 signaling [60].

### 2.4. The Role of T Cells in Malaria

Blocking immune response prevents T cells from exhaustion, increasing the T cell effector function and allowing interactions between immune cells [61]. The balance of the immune response depends on the optimal T cell activation to the successful resolution of the microbial infections, as well. The negative regulators of T cell activation of the cytotoxic T-lymphocyte-associated protein 4 (CTLA-4) and programmed cell death (PD-1)/programmed death-ligand (PD-L), damps responses during chronic infections [62]. The CTLA-4 and PD-1/PD-L1 inhibitory pathways independently regulate host resistance to plasmodium-induced acute immune pathology [62]. A multimeric form of PDL2 fused with the Fc region of immunoglobulin (PDL2–Fc) attenuates lethal malaria infection [63]. Likewise, a combined blockade of the inhibitory molecules PDL1 and lymphocyte activation gene 3 protein (LAG3), accelerate the clearance of acute non-lethal *Plasmodium yoelii* malaria [64]. Antibody-mediated triggering of the T cell co-stimulatory molecule OX40 signaling also improved parasite clearance in malarial infections [65]. Additionally, blocking T cell immunoglobulin and mucin domain-containing protein 3 (TIM3) signaling with an antibody, resulted in accelerated parasite clearance and reduced symptoms of cerebral disease in *Plasmodium berghei*-infected mice [66]. Antibody-mediated blockade of either CTLA4 or PDL1, but not the PD-1/PD-L2, increased the incidence of cerebral malaria in resistance mice in *Plasmodium berguei* ANKA (PbA) infection [62]. Thus, checkpoint blockade could complement malarial drugs to generate long-term immunity and protect populations in endemic areas.

Diseases caused by intracellular parasites, such as Plasmodium, depend on the IFN-γ-producing Th1 cells to activate dendritic cells and macrophages for capturing, antigen presentation, and for killing pathogens [67]. However, the Th1 cells produce inflammatory cytokines, damaging tissues. IL-10 is a regulatory molecule in malaria, which could prevent excessive inflammation [68]. Studies from mice infected with malaria, IL-10 dampen the inflammatory response, prevent tissue damage, and protect animals [69]. Conversely, the IL-10 anti-inflammatory effect was associated with severe anemia and high parasitemia in experimental malaria [70,71]. Therefore, IL-10 protects host tissues against damaging inflammation, but by doing so, it can foster parasite growth [72].

The splenic plasmacytoid dendritic cells might act as a Trojan horse and carry the live Plasmodium within it. Rapamycin treatment reverses it by inducing an autophagic flux shift of the aggressive Th1 immune pathway, which is reported to incur host damage to a better well-balanced cytokine profile Th2 pathway (increase in IL10/TNF-α, regulatory T cell Treg/Th17) [73]. The IL-10 also has a role in cross-reactive memory response. Mice primed against *Plasmodium chabaudi (Pcc)* and infected with and *Plasmodium Berghei* ANKA (PbA) present high levels of IFN-γ and IL-10. Blocking IL-10 receptor increases the activated CD4^+^ and γδ T cells and the IFN-γ production in response to PbA, with a reduction of parasitemia [74]. Although IFN-γ protects against malaria infection, it has been linked to cerebral malaria and immunopathology [75,76].

Leukotriene B4 (LTB4) was found to be increased in serum mice with cerebral-malaria-induced IFN-γ through dendritic cells [77,78]. Treatment with aspirin caused an overproduction of the omega-6 arachidonic acid (AA) metabolite LTB4 [79]. Hence it might be associated with overproduction of IFN-γ in experimental and human cerebral malaria [80]. Treatment with the AA metabolite leukotriene C4 (LTC4) induced erythrocyte cell death [81]. So LTC4 might induce a clearance of pRBC against malaria infection; contrariwise, the excessive erythrocyte cell death might cause anemia [80].

Naturally acquired immunity to malaria requires both innate and acquired immune responses. Antibodies generated to recognize Plasmodium antigens are lost without the development of immunological B cell memory. Infections during pregnancy might expose the fetus to microbial antigens triggering inflammation and fetal immune response, affecting the B cell. It could prime the infant immune system and its response against a challenge might be augmented [82]. Ly and Hansen reviewed the importance of a proper response and the generation of a long-term B cell memory in malaria infection [83].

### 2.5. Cerebral Malaria and Inflammation

Sequestration of pRBC within the brain microvasculature is a hallmark of cerebral malaria. *Plasmodium falciparum*-pRBC isolated in children with complicated malaria, increased binding to brain microvascular endothelial cells, with better adhesion compared to uncomplicated malaria cases. The *var* gene transcripts predict to bind host endothelial protein C receptor and intercellular adhesion molecule 1 (ICAM-1) [84]. Post mortem analysis showed that children who died of malaria had sequestered pRBC in the retinal microvessels [85]. Binding of pRBC to host endothelial protein C receptor (EPCR) inhibits its interaction with activated protein C, leading to alterations in coagulation, inflammation, and endothelial barrier [86,87,88,89]. Reinforcing the idea that cerebral malaria pathology is based on increased cytoadherence of pRBC in the brain microvasculature [84]. Malaria PbA infection increased lipid peroxidation, and the release of TNF and-α IFN-Υ, and capsazepine (antagonist of transient receptor potential channels, of the vanilloid subtype—TRPV1)-treated group had lower levels of these products [90]. The TRPV1 knockout (KO) exhibited lower concentrations of plasma and brain cytokines, especially TNF-α and IL-6, less cerebral swelling with higher levels of junctional adhesion molecule A, and claudin-5, in their brain vasculature. The TRPV1 KO mice presented less neuronal damage, and they were protected against Plasmodium-induced mortality and morbidity, but the Plasmodium load was not affected [91].

A new approach based on the analysis of the genetic effects of inflammatory response gene variants might offer a new rationale for use, in the prevention and management of severe malaria in infected people who develop severe clinical syndromes. Some inflammatory response genes can be associated with malaria, like TNF-α, NOS2, Interferon-alpha/beta receptor alpha chain gene (IFNAR1), heme oxygenase 1 (HMOX1), TLRs, CD36, and CD40LG. Nevertheless, we must consider that pro-inflammatory genetic variants in early infection stages are useful in resolving malaria, but at later stages these genetic variants relates to enhanced vulnerability to organ damage, resulting from inflammation [35].

## 3. Inflammation and Fatty Acids

### 3.1. Omega-9

Non-esterified fatty acids (NEFA) play many essential functions in living organisms, but they can also be toxic to cells. MUFA oleic acid, an omega-9, can be pro-inflammatory or anti-inflammatory, depending on its dose or concentration. The omega-9, oleic acid, is a PUFA with the first double bond on the ninth carbon from the terminal methyl end (Figure 1). Our group has given contributions to this field. Increased plasma concentrations of this omega-9 and other fatty acids increase during diseases. Disease severity in sepsis and leptospirosis can be associated with high plasma levels of NEFA and low blood albumin concentration, denoting NEFA toxicity [92].

Omega-9 induced acute lung injury in experimental models. Increased omega-9 (oleic acid) plasma concentrations cause lung injury by interfering with sodium transport controlled by Na/K-ATPase, leading to acute respiratory distress syndrome (ARDS). Dosage of omega-9 completely inhibited lung Na/K-ATPase activity-induced lung edema and leukocyte accumulation, lipid body formation in leukocytes, and the production of the omega-6 AA metabolites, such as LTB_4_ and prostaglandin E_2_ (PGE2) [93]. The omega-9 intratracheal injection caused edema, cell migration and activation, production of lipid mediators, and cytokines on a mechanism dependent on intracellular signaling triggered by the mitogen-activated kinase (MAP) ERK1/2 activation, because of pharmacological inhibition of ERK1/2 phosphorylation blocked neutrophil migration, edema, lipid body formation, and IL-6 production [94].

**Figure 2:**
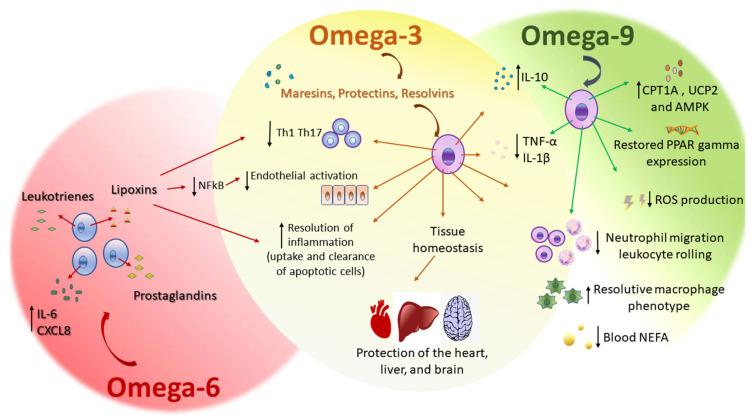
Effects of omega-6, omega-3, and omega-9 on inflammation in host cells. IL—interleukin, CXCL8—C-X-C motif chemokine ligand 8, NFκB nuclear factor kappa B, Th—T helper cells, IL—interleukin, TNF-α—tumor necrosis factor alpha, CPT1A—carnitine palmitoyltransferase 1A, UCP2—uncoupling protein 2, AMPK—5′ adenosine monophosphate-activated protein kinase, ROS—reactive oxygen species, and NEFA—non-esterified fatty acid.

Plasma NEFA increases in severe systemic inflammatory response syndrome as sepsis, a syndrome characterized by both inflammatory and metabolic alterations, including massive cytokine production, oxidative stress, lipid metabolism disturbances, and organ dysfunction. Although omega-9 (oleic acid) can cause inflammation unexpectedly, 24 h after either intravenous or intragastric administration of the omega-9, the primary NEFA plasma levels were dose-dependently reduced. This effect of omega-9 on the decrease of blood NEFA levels can account for the health benefits of unsaturated fatty-acid-enriched diets, such as the MedDiet, reducing the risk of cardiovascular diseases and cancer [92]. The MedDiet is based on olive oil consumption. Omega-9, oleic acid, is the main component of olive oil. The supplementation for 14 days with this omega-9 ameliorated clinical outcome, increased the survival rate, decreased liver and kidney injury, and diminished NEFA plasma levels in mice subjected to the experimental sepsis model of cecal ligation and puncture (CLP). Omega-9 was effective in diminishing reactive oxygen species (ROS) production and NEFA plasma levels. The beneficial effect of omega-9 supplementation was followed by an increase in translation of the enzyme carnitine palmitoyltransferase IA (CPT1A) and uncoupling protein 2 (UCP2) liver-enhanced expression. These phenomena were compatible with the finding that the decrease in 5′ AMP-activated protein kinase (AMPK) expression found in the liver of septic mice was prevented by omega-9. We suggest that a pretreatment with omega-9 decrease NEFA concentration and increase CPT1A and UCP2 and AMPK levels, and a decrease ROS production (Figure 2). The prevention of metabolic dysfunction by omega-9 has a beneficial role in sepsis, supporting the benefits of diets high in MUFA [95].

Besides the role of omega-9, oleic acid, supplementation on metabolic dysfunction during sepsis, omega-9 treatment increased levels of the anti-inflammatory cytokine IL-10 and decreased levels of the pro-inflammatory cytokines TNF-α and IL-1β, in the peritoneum of septic mice with restored corticosterone levels. The anti-inflammatory role of the omega-9 included a significant inhibitory effect on neutrophil migration, from circulation to the peritoneal cavity and on leukocyte, rolling on the endothelium. Omega-9 also increased bacterial elimination in the peritoneal lavage, suggesting a direct effect on bacteria and an indirect one, on improving the ability of inflammatory leukocytes to fight against infection. Furthermore, omega-9 restored liver and adipose tissue expression of the transcription factor peroxisome-proliferator-activated receptor gamma (PPARγ) expression in septic animals (Figure 2). PPARγ controls inflammation by both negatively modulating the production of pro-inflammatory cytokines and enhancing the synthesis of anti-inflammatory cytokines during sepsis [96]. PPARγ also protected the brain against microvascular dysfunction in sepsis, including diminished leukocyte/endothelial cell interaction and increased functional capillary density in the brain, improving cerebral perfusion [97]. The beneficial anti-inflammatory role of omega-9 in sepsis possibly can occur through a mechanism dependent on PPARγ expression and can join the many described beneficial properties of NEFA-enriched diets in inflammatory diseases [98].

Inflammation associated with metabolic dysregulation is also a common feature in aging. Plasma saturated, PUFA, MUFA, and circulating TNF-α and IL-6 concentrations increased with age, whereas IL-10 and TGF-β1 concentrations decreased with age. Higher levels of the saturated fatty acids C18:0 and C24:0 are linked to lower TGF-β1 concentrations, and higher C16:0 are linked to higher TNF-α concentration. C16:0 also induces the production of cytokines in vitro. Monocytes incubated with C16:0 produced pro-inflammatory cytokines during phorbol myristate acetate (PMA)-induced differentiation. In contrast, the effect of omega-9 was opposite, priming a macrophage phenotype associated with the resolution of inflammation [99].

### 3.2. Omega-3

Omega-3 are PUFA with the first double bond on the third carbon from the terminal methyl end found (Figure 1) in fish and flaxseed oils. The major fatty acids of the omega-3 family in the fish oil are EPA and DHA. ALA is found in flaxseed oil. Omega-3 protects the heart, liver, and brain [100]. The substrates that generate active metabolites produced by COX, lipoxygenases, and cytochrome P450 monooxygenases, are precursors of the anti-inflammatory signaling molecules eicosanoids. These are players in the control of inflammation [101] and display a wide range of beneficial effects in humans and animals. Omega-3 controls inflammation and tissue homeostasis [102]. Some studies value the link between omega-3 and low-grade inflammation, because omega-3 might displace the eicosanoid metabolism to the formation of mediators with low or no inflammatory activity, decreasing endothelial activation [101]. Diet supplementation with fish oil or omega-3 has a beneficial effect on skin inflammation. In UV-irradiated skin, EPA altered the lipidome, diminishing the production of pro-inflammatory lipids, and DHA inhibited the migration of Langerhans cells [103]. DHA is a substrate for the generation of mediators, as maresins are also involved in tissue regeneration. Macrophages are crucial in producing these mediators and regulating inflammatory response, initiating and promoting its resolution. These mediators promote the uptake and clearance of apoptotic cells, accelerating the resolution of inflammation, culminating in tissue repair and regeneration [104].

Besides EPA and DHA, another important omega-3 PUFAs has recently emerged as specialized pro-resolving mediators like resolvins, maresins, and protectins. DPA, a 22:5 long-chain PUFA [105], can be found with EPA and DHA in fish oils in a percentage of: EPA 10–13%, DPA 2–5%, and DHA 9–11% [106,107]. It also is an elongated metabolite of EPA and is an intermediary metabolite between EPA and DHA [107]. DPA products also have both tissue-protective and anti-inflammatory responses [108]. The protectins derived from DPA reduce human neutrophil–endothelial cell interactions, neutrophil chemotaxis and recruitment, and increased macrophage phagocytosis [108,109]. During sterile inflammation, DPA protectins carry potent leukocyte directed actions, reduce the production of inflammatory cytokines, including monocyte chemoattractant protein-1 (MCP-1/CXCL-2) [110]. Maresin 1 stimulated macrophage phagocytosis and clearance of human apoptotic neutrophils, similar to DHA-derived Maresin 1 [111]. It indicates that biological effects displayed by n-3 fatty acids that has been attributed to EPA and DHA might have be due to DPA as well [112]. DPA even showed a stronger anti-inflammatory effect than EPA and DHA, in an ulcerative colitis model due to its easier incorporation into inflammatory cells [113].

A switch from acute to chronic inflammation can occur when the resolution of inflammation is disrupted, as in chronic heart failure (CHF), where T lymphocytes play a critical role. The omega-3-derived specialized pro-resolving lipid mediator resolvin is low in plasma of patients with CHF, because of low expression in 15-lipoxygenase on their leukocytes. CD8^+^ and CD4^+^ T cells were unresponsive in CHF patients. They also show a decrease in the resolving 1 receptor (GPR32), which might be linked to the progression of chronic inflammation, raising the role of omega-3-derived lipids and their signaling pathways as potential targets to CHF treatment [114].

Lipoxygenase omega-3 metabolite resolvin play a role in *Trypanossoma cruzi* infection, causing a decrease in inflammation and fibrosis in chronic heart disease in mice, consequently increasing the host survival. Resolvin D1 also effective regulates the cell activation in patients with Chagas disease. During *Leishmania* spp. infections, resolvin D1 control the cutaneous manifestation of diseases [115]. During infection, resolvin enhanced phagocytic clearance of bacteria, downregulated inflammation caused by infection, accelerated resolution of inflammation by promoting neutrophil apoptosis, besides modulating neutrophil migration ability and increased survival. Resolvin was adjuvant to ciprofloxacin and vancomycin in inducing antibacterial effect, indicating a promising new strategy in managing infectious disease [116].

Omega-3 effects are potentiated by non-steroidal anti-inflammatory drugs (NSAID) like aspirin. Their combination promotes the generation of bioactive lipids that better control inflammation. Mice treated with omega-3 and aspirin produce those metabolites. For instance, aspirin stimulates human endothelial cells with upregulated COX-2, to convert C20:5 omega-3 to 18R-hydroxyeicosapentaenoic acid (HEPE) and 15R-HEPE, which are inhibitors of neutrophil transendothelial migration and infiltration. These are the benefits of omega-3 supplementation, which is crucial in modulating inflammation, cancer, and vascular diseases [117].

### 3.3. Omega-6

The omega-6 AA (Figure 1) is the main fatty acid present in the membrane phospholipids of cells responsible for causing inflammation. AA is a substrate for COX and lipoxygenase, and these lead to potential inflammatory mediators called eicosanoids, like prostaglandins and leukotrienes (Figure 2), respectively [118]. Omega-6 are the precursors of many pro-inflammatory signaling molecules that trigger inflammation [101].

Dietary fatty acids alter immune responses caused by a respiratory infection in pulmonary fibroblasts and bronchial epithelial cells. AA cause IL-6 and CXCL8 release, and it is even higher with AA and the bacterial compound lipoteichoic acid (LTA) together. The production of cytokines by pulmonary fibroblasts depends on prostaglandin, and synergism depend on p38 mitogen-activated protein (MAP) kinase signaling. The high content of dietary omega-6, such as in obese patient diet, could lead to more severe inflammation [119].

Lipoxins (LX) are also produced from AA by lipoxygenase activity in the inflammatory site and present both anti-inflammatory and pro-resolving bioactions [120]. LX reduced neutrophil migration in vitro and increased the neutrophil clearance in vivo [121]. Lipoxin A4 (LXA_4_) regulates leukocyte trafficking and responses [122], it likewise modulates the activation of vascular, smooth muscle, and epithelial cells [123], and reduces organ fibrosis [124]. LXA4 also attenuates nociception through actions on astrocyte-expressed lipoxin receptors, reducing pain [125]. Despite LX anti-inflammatory properties and pro-resolutive effects [126], they do not impair host defense against infection [127]. LX have beneficial effects in infectious diseases caused by *Mycobacterium tuberculosis* infections, because they promote necrotic death of infected macrophages and inhibit the initiation of cell-mediated immunity [128,129]. In *Trypanosoma cruzi* infection, 15-epi-LXA4 reduced parasitemia and increased survival [130]. Conversely, in *Toxoplasma gondii* infection, LX reduced mortality but increased parasite load [131]. In sepsis, LX increased survival by reducing systemic inflammation [132]. The binding of LXA4 to its receptor ALX can modulate the expression of adhesion molecules through inhibition of the NFκB pathway in endothelial cells (Figure 2) [133,134,135].

### 3.4. Omega-3 Versus Omega-6

Common sense believes dietary intake of the omega-6 AA or its precursor linoleic acid (LA) potentiates inflammation because the synthesis of the pro-inflammatory AA metabolites prevail over the production of anti-inflammatory AA metabolites or resolutive mediators (Figure 2). Some references, however, have shown there is no correlation. Nevertheless, the competition of both substrates and high omega-6 diet content would favor the production of pro-inflammatory and an inhibition of the anti-inflammatory and pro-resolutive effect of omega-3 [118].

An alternative use of NSAID inhibits the formation of AA metabolites because of their role on COX inhibition, and therefore, its deleterious side effects would be a diet rich in EPA/DHA. Supplementation with fish oil inhibits inflammatory cytokines, such as TNF-α and IL-1β and pro-inflammatory and pro-aggregatory eicosanoids such as thromboxane-2 and PGE2. Eating omega-6 seed oils have the potential to increase those phenomena and omega-3 suppress inflammation. A balance, favoring the enrichment of a diet with omega-3 might profit numerous chronic inflammatory diseases, such as rheumatoid arthritis, atherosclerosis, dyslipidemia, diabetes, obesity and heart failure [136], sepsis, and malaria.

Supplementation of an appropriate omega-6/omega-3 ratio modulated T helper (Th) cells and Treg cells that are involved in the exacerbated immune response related to the progression of sepsis. In an experimental model of sepsis fed with a diet rich in fish oil with an omega-6/omega-3 PUFA ratio of 2:1, decreased inflammatory mediator production in the plasma and peritoneal lavage (Figure 2), compared to omega-6/omega-3 PUFA ratio of 7:1 rich diet. This was followed by a decrease in Th1, Th2, and Th17 cells (Figure 2), and a reduction of neutrophil infiltration. A diet rich in omega-6/omega-3 PUFA ratio of 2:1, upregulated the transcription factor PPARγ. The activation of PPARγ might be the molecular mechanism involved in which a fish oil-enriched diet maintains balanced Th polarization, mitigates inflammation, and alleviates exacerbated inflammation in sepsis [96].

Besides the omega-3 effect on T cells, dietary omega-3 long-chain PUFA control inflammation in mice by modulating dendritic cell function during experimental autoimmune uveitis (EAU). An omega-6 long-chain PUFA diet has not shown the same effect. Transfer of dendritic cells from mice fed omega-3 long-chain PUFA diet to mice with EAU decreased the T cell proliferation and the production of IFN-γ and IL-17 by T cells, mitigating the disease development. Splenocytes were isolated from the mice and cocultured with CD4^+^ T cells isolated from mice with EAU [137]. Omega-3 long-chain PUFA seem to have a molecular target in T cells, the T-bet and RORγt, Th1- and Th17-related transcription factors, respectively. T-bet and RORγt transcription was diminished in the retina and lymph node cells of mice fed with rich omega-3 long-chain diet, but not with an omega-6 long-chain one [138].

## 4. Fatty Acids and Malaria

### 4.1. Fatty Acid Metabolism of the Parasite as a Target of Antimalarial Compounds

The malaria parasite goes through several morphological stages during its life cycle [26]. The intense proliferation demands nutrients, including fatty acids. Parasites obtain fatty acids by two pathways, scavenging from the vertebrate host and mosquito vector or by producing them through the fatty acid synthase type II (FAS-II) [139,140]. This enzyme was expressed in the mosquito midgut and liver-stage parasites [140], the blood-stage did not require the FAS-II [141,142,143]. *P. falciparum* FAS-II knockout parasites failed to complete sporogony, revealing that the FAS-ll pathway is essential for the human infection phase [140]. Mostly due to the differences in FAS-I and the Plasmodium FAS-II, the last emerges as a promising drug target, especially for the clinically silent liver state infection. In this regard, synthesized 2-, 5-, 6-, and 9-hexadecanoic acids (HDAs) were developed. *Plasmodium falciparum* blood stages 5-HAD, displayed the highest antiplasmodial activity, and 2-HDA was the most significant inhibitor of the FAS II enzymes [33].

A series of 2-alkynoic fatty acids (2-AFA) with chain lengths between 14 and 18 carbon atoms were tested against *Plasmodium falciparum* and *Plasmodium berghei*. Treatment with 2-ODA and 2-TDA was effective in treating infection and killing parasites in hepatic cell cultures. Among them, 2-ODA was the most effective inhibitor, with the three critical enzymes in the FAS II elongation pathway being even more potent than primaquine [144].

Four fatty acids from lichens, the evernic, vulpic, psoromic, and (+)-usnic acids, act indirectly via binding to allosteric sites on the protein surface of the FAS-II enzymes (PfFabI, PfFabG, and PfFabZ elongation enzymes), thus, having an antiplasmodial effect [145].

Fatty acid biosynthesis is an interactive process, beginning with the condensation of acetyl-CoA, with a growing fatty acid chain. *Plasmodium falciparum* enoyl acyl carrier protein (ACP) reductase (PfENR) is responsible for the final step in each fatty acid synthesis cycle [146]. Fabl is an enzyme that catalyzes the last step of biosynthesis in the FAS-II pathway and is also known as an enoyl-ACP reductase. In the malaria *Plasmodium berghei* mouse model, the use of triclosan, a potent inhibitor of FabI, actively suppressed parasitemia [147]. Additionally, triclosan shows activity against Plasmodium asexual blood-stage parasites and shows efficacy in vivo, whereas FabI-deficient *Plasmodium berghei* sporozoites failed to complete liver-stage development in vitro [141].

Turkish marine sponge *Agelas oroides*, produce a vast diversity of pyrrole and imidazole alkaloids [148], yielding a variety of pure metabolites that have inhibitory effects against *Plasmodium falciparum* enoyl-ACP reductase (PfFabI) enzyme [149].

A rhodanine (2-thioxothiazolidin-4-one) class of inhibitory compounds of enoyl acyl carrier protein (ACP) reductase inhibited Plasmodium growth in RBC cultures [150], even in chloroquine-sensitive *Plasmodium falciparum* [151].

The continuous growth of parasite resistance requires novel modes of action. Fatty acids have also been used on insecticide new formulation strategies. An insecticide comprising straight-chain octanoic, nonanoic, and decanoic saturated fatty acids (C8, C9, and C10) called C8910, displayed insecticidal effects on different strains of *Anopheles funestus* [152]. Supplemented birds with olive oil and vitamin E increased the transmission to the vector, indicating that vertebrate host oxidative status influences parasite transmission to the vector [153].

### 4.2. Omega-3, Omega-6, and Malaria

Since 1957, Godfrey has shown that diet alterations might influence the development of malaria disease in mice. The *Plasmodium berghei* infection was utterly suppressed in the mice, when given a high cod liver oil diet and also in those given vitamin “E” [154]. Feeding mice 4 weeks before inoculation with *Plasmodium yoelii* with the fish oils (menhaden, anchovy, or salmon) in a vitamin E-deficient diet, actively protected against malaria in mice, reducing parasitemia and improving survival [155,156]. Mice supplemented with SFA, EPA, and DHA fortified with alfa-tocopherol and that infected with *Plasmodium yoelii*, reduced parasitemia and improved survival rates [156,157].

Mice fed with fish oil diets rich in EPA and DHA with vitamin E did not survive, but when fed without vitamin E they had considerably improved survival, and the effect lasted for months [158]. Yet, C-18 fatty acids (omega-9 oleic acid, elaidic acid, and linoleic acid) displayed a considerably inhibitory activity, both in infected cells and in free parasites *Plasmodium falciparum*, *Plasmodium vinckei petteri*, and *Plasmodium yoelii*. Vitamin E addition to a culture of trophozoite-infected cells did not reverse the inhibitory effects of fatty acids [159].

EPA, DHA, and AA enhanced neutrophil antimalarial activity with increased superoxide radical generation in response to *Plasmodium falciparum* [160]. Fatty acids stimulate the generation of free radicals by leukocyte to kill *Plasmodium falciparum*. Patients with active *Plasmodium falciparum*-positive malaria with lower concentrations of lipid peroxides and nitric oxide allow an infection to persist, whereas elevated levels of EPA might indicate an attempt to overcome the infection [161]. The animal malaria model treated with DHA showed a decrease in the duration of malaria symptoms [162]. Both, treatments with linolenic acid (omega-3) and linoleic acid (omega-6), showed a potent inhibition of the parasitemia in *Plasmodium berghei* infection [163].

Diet supplementation with omega-3 makes the host response more prompt to a resolutive response, accelerating the resolution of inflammation. Nevertheless, the administration of the omega-6 metabolite LX also negatively modulates inflammation by shifting to a more anti-inflammatory phenotype. The lipoxin epimer 15-epi-LXA4 and LXA4 itself, as discussed before, have numerous anti-inflammatory properties [164,165,166]. The administration of 15-epi-LXA4 reduced brain inflammation by inhibiting IL-12 production, and accumulation of IFN-producing cells in the brains of infected mice decreases lymphocyte infiltration and enhances survival [36]. Similarly, treatment with LXA4 reduced vascular occlusion and reduced *Plasmodium berghei*-infected erythrocytes adhered to brain vasculature, besides an improved functional capillary density [49]. Moreover, LXA4-induced heme oxygenase 1 (HO-1), an enzyme associated with tissue protection [167], in endothelial dysfunction in vivo [168] and in vitro [169], which are essential to the prevention of severe malaria development. HO-1 is related to the inhibition of adhesion molecules preserving endothelial functions and BBB integrity [170,171]. Additionally, HO-1 in brain tissue was associated with mouse survival and decreased cerebral edema [172].

Lung injury in severe malaria is a concern. Neutrophil accumulation and alveolar-capillary membrane alterations cause lung damage, leading to physiological dysfunction. In *Plasmodium berghei* infection, pretreatment with LXA4, prevented lung edema and preserved lung morphology and function, compared to nontreated *Plasmodium berghei*-infected mice. During *Plasmodium berghei* infection, pretreatment or posttreatment with LXA4 alleviated lung neutrophil accumulation, reduced alveolar collapse, thickening and edema, and decreased the production of CXCL1 in plasma [173]. LXA4 also affected neutrophil migration in vitro, stimulated by plasma from mice infected with *Plasmodium berghei*, by disrupting neutrophil cytoskeleton that inhibits F-actin polarization. In conclusion, LXA4 exerted therapeutic effects in malaria-induced ALI by inhibiting lung dysfunction [173].

A healthy diet stimulating the increase of the daily consumption of omega-3 has been broadly discussed as a link between increased consumption of omega-3 to a less inflammatory phenotype. It is valid for malaria as well, but interestingly and unexpectedly, the administration of a high-fat saturated diet to mice for just 4 days dramatically mitigates *Plasmodium berguei* ANKA infection. Oxidative stress seems to be involved in biochemical mechanisms. Products of fatty acid β-oxidation impact Plasmodium survival in hepatocytes, even transient dietary changes impact the *Plasmodium berguei* ANKA infection and the development of the disease [174].

## 5. Concluding Remarks

The MedDiet has beneficial effects on cardiovascular diseases, diabetes, metabolic syndrome, certain types of cancer, and inflammatory and infectious diseases [1,5,98,175,176]. MedDiet consists of a large number of bioactive ingredients, including high content of olive oil and fish rich in omega-3 and omega-9. Both fatty acids have anti-inflammatory [2] and pro-resolutive effects [104], acting pharmacologically and on biochemical targets [177]. The omega-3 and omega-9 show metabolic and immune regulatory effects, modulating intracellular pathways and transcription factor activation. The lipid mediators generated through omega-3 or omega-6 metabolism have a critical role in controlling inflammatory, infectious, and parasitic diseases [98], including malaria. Besides omega-3 and omega-9, saturated fatty acid and omega-6 have emerged with potential beneficial effects on experimental malaria by controlling excessive inflammation, impacting malaria infection development, and clinical outcome. Dosing the amount and type of fatty acid in MedDiet is critical to seal the inflammatory, infectious, and parasitic disease progression. Further controlled studies will unearth the potential benefits of increased consumption of specific fatty acid components of the MedDiet.

## Figures and Tables

**Table 1:** Daily nutritional suggested composition for the experimental MedDiet (2240 kcal/d).

Nutrients	Amount
Carbohydrate (g)	194.0
Fiber (g)	41.1
Protein (g)	87.3
Fat (g)	106.0
SFA (g)	20.9
MUFA (g)	52.5
PUFA (g)	24.8
Cholesterol (mg)	289.7
Alcohol (g)	11.1
MUFA to SFA ratio	2.7
Omega-3 (g)	5.9
Omega-6 (g)	18.7
Cholesterol (mg)	255.8
Beta carotene (μg)	3039
Vitamin A (RE)	2161.6
Vitamin D (μg)	3.2
Vitamin E (mg)	29.3

This table has been modified from other publications [13,14,15,16]. SFA—saturated fatty acid, MUFA—monounsaturated fatty acid, PUFA—polyunsaturated fatty acid.
